# Value of variation of end-tidal carbon dioxide for predicting fluid responsiveness during the passive leg raising test in patients with mechanical ventilation: a systematic review and meta-analysis

**DOI:** 10.1186/s13054-022-03890-9

**Published:** 2022-01-14

**Authors:** Haijun Huang, Chenxia Wu, Qinkang Shen, Yixin Fang, Hua Xu

**Affiliations:** Department of Emergency, The First Affiliated Hospital of Zhejiang Chinese Medical University, Hangzhou, 310018 Zhejiang China

**Keywords:** Fluid responsiveness, End-tidal carbon dioxide, Mechanical ventilation, Meta-analysis

## Abstract

**Background:**

The ability of end-tidal carbon dioxide (ΔEtCO2) for predicting fluid responsiveness has been extensively studied with conflicting results. This meta-analysis aimed to explore the value of ΔEtCO2 for predicting fluid responsiveness during the passive leg raising (PLR) test in patients with mechanical ventilation.

**Methods:**

PubMed, Embase, and Cochrane Central Register of Controlled Trials were searched up to November 2021. The diagnostic odds ratio (DOR), sensitivity, and specificity were calculated. The summary receiver operating characteristic curve was estimated, and the area under the curve (AUROC) was calculated. *Q* test and *I*^2^ statistics were used for study heterogeneity and publication bias was assessed by Deeks’ funnel plot asymmetry test. We performed meta-regression analysis for heterogeneity exploration and sensitivity analysis for the publication bias.

**Results:**

Overall, six studies including 298 patients were included in this review, of whom 149 (50%) were fluid responsive. The cutoff values of ΔEtCO2 in four studies was 5%, one was 5.8% and the other one was an absolute increase 2 mmHg. Heterogeneity between studies was assessed with an overall *Q* = 4.098, *I*^2^ = 51%, and *P* = 0.064. The pooled sensitivity and specificity for the overall population were 0.79 (95% CI 0.72–0.85) and 0.90 (95% CI 0.77–0.96), respectively. The DOR was 35 (95% CI 12–107). The pooled AUROC was 0.81 (95% CI 0.77–0.84). On meta-regression analysis, the number of patients was sources of heterogeneity. The sensitivity analysis showed that the pooled DOR ranged from 21 to 140 and the pooled AUC ranged from 0.92 to 0.96 when one study was omitted.

**Conclusions:**

Though the limited number of studies included and study heterogeneity, our meta-analysis confirmed that the ΔEtCO2 performed moderately in predicting fluid responsiveness during the PLR test in patients with mechanical ventilation.

## Introduction

Fluid resuscitation is recommended and widely used as the first-line resuscitative therapy for all patients presenting with acute circulatory failure [1]. Although the volume status of a shocked patient is recovered, evidence suggests that inappropriate administration of fluids has deleterious effects such as volume overload, systemic and pulmonary edema, and limitation of oxygen diffusion to tissues, thereby leading to increased tissue hypoxia [2–4]. Therefore, it is important to obtain reliable information about fluid responsiveness in patients having a circulatory failure in the intensive care unit. However, clinicians are often faced with inaccurate, nonspecific information to guide their treatment.

Previous studies have shown that some parameters may be related to volume status. The traditional static parameters, such as intrathoracic blood volume index, pulmonary wedge pressure, and central venous pressure, have been proved not related to patient volume status [5, 6]. Hemodynamic parameters, such as pulse pressure variation and stroke volume variation, may better predict fluid responsiveness. However, the evaluation of these parameters requires invasive procedures and special monitoring equipment, limiting their clinical application [7].

Passive leg raising (PLR) induces a rapid and reversible increase in preload through an increase in venous return mimicking fluid administration. This maneuver has been demonstrated to predict fluid responsiveness [8, 9], However, a fast response and direct measurement technique of the effect on cardiac output is needed. Nevertheless, the need to monitor cardiac output with echocardiography, arterial pulse contour analysis, bioreactance, or esophageal Doppler is unachievable or inconvenient under many clinical conditions.

End-tidal carbon dioxide (EtCO2) is the partial pressure of carbon dioxide (PCO2) in the exhaled air measured at the end of expiration. Measurement of EtCO2 using capnography provides a noninvasive estimate of cardiac output during cardiac arrest and can therefore be used to monitor the quality of cardiopulmonary resuscitation and predict return of spontaneous circulation [10–12].In recent years, the variation of EtCO2 (ΔEtCO2) during passive leg raising (PLR) test or fluid challenge has been considered as a tool to help guide fluid resuscitation [13–19], however, the results conflicting. Physiologically, EtCO2 depends on three variables: tissue CO2 production, pulmonary blood flow (i.e., cardiac output), and alveolar ventilation [20]. Thus, EtCO2 may accurately reflect cardiac output when ventilator parameters and CO2 production are constant. This correlation has been tested in experimental [21] and clinical [22] studies. Since PLR is a short maneuver, these two factors are assumed to be constant and therefore, EtCO2 should mainly reflect changes in CO, it is theoretically possible to assess fluid responsiveness after PLR test.

In this systematic review and meta-analysis, the test characteristics of ΔEtCO2 were summarized as a predictor of fluid responsiveness during PLR test in patients with mechanical ventilation to elucidate their diagnostic performance further and provide information for the detection of fluid responders.

## Materials and methods

This meta-analysis was conducted according to the Preferred Reporting Items for Systematic Reviews and Meta-analyses guidance [23].

### Registration and protocol

This meta-analysis was registered on PROSPERO (CRD42021284241).

### Search strategy

Relevant studies up to November 2021 were searched in the PubMed, Embase, and Cochrane Library databases with the following terms and their combinations: “fluid therapy OR fluid responsive OR volume responsive,” “end tidal carbon dioxide OR end-tidal carbon dioxide OR EtCO_2_” and “mechanical ventilation OR ventilated.” All scanned abstracts, studies, and citations were reviewed. Moreover, references of the retrieved manuscripts were also manually cross-searched for further relevant publications.

### Selection criteria

The inclusion criteria were as follows: (1) studies on patients receiving mechanical ventilation; (2) studies with PLR-induced increase in EtCO2 as the index test; (3) studies with a CO monitoring for the diagnosis of fluid responsiveness; (4) studies published with full-text in any language; (5) studies providing sufficient data for constructing 2-by-2 tables, including true positive (TP), false positive (FP), true negative (TN), and false negative (FN). The exclusion criteria were as follows: (1) studies that used the same population or overlapping database and (2) studies on animal models.

### Data extraction and quality assessment

All the available data were extracted from each study by two investigators independently according to the aforementioned inclusion criteria, and any differences were resolved by discussion with a third investigator. The following data were collected from each study: (1) basic characteristics of studies, including first author name, publication year, country where the research was performed, selected patients, gender, mean age, number of patients, tidal volume, index test device for the EtCO2, reference standard measurement for cardiac output monitoring, reference standard threshold, and reference standard device; (2) diagnostic performance, including cutoff value, sensitivity, specificity, area under the receiver operator characteristic curve (AUROC), TP, FP, FN, and TN. The quality of included studies was scored independently by two reviewers using the revised Quality Assessment of Diagnostic Accuracy Studies (QUADAS-2) criteria [24]. The quality of studies was assessed using RevMan 5.4.

### Statistical analysis

All analyses were performed using the Stata 16.0 software (Stata Corp., College Station, TX, USA). The bivariate meta-analysis model was employed to summarize sensitivity, specificity, positive likelihood ratio, negative likelihood ratio, and diagnostic odds ratio (DOR) [25, 26]. The sensitivity and specificity of each included study were used to plot the summary receiver operator characteristic (SROC) curve and calculate the area under the SROC curve (AUC). Diagnostic power was good, moderate, and poor if the AUC was more than 0.8, between 0.7 and 0.8, and less than 0.7, respectively [27]. As publication bias is a concern for meta-analyses, the Deeks’ funnel plot asymmetry test was used, with *P* < 0.10 indicating statistical significance [28]. If publication bias was present, a sensitivity analysis of DOR was performed to explore why.

Spearman’s correlation coefficient between the logit of sensitivity and logit of 1-specificity was calculated to determine any threshold effect; A strong positive correlation would suggest threshold effect [29]. The between-study heterogeneity was evaluated using *Q* test and *I*^2^ statistics. A *P* value less than 0.10 for the *Q* test or *I*^2^ value ≥ 50% indicated substantial heterogeneity. A fixed effects model was used if no heterogeneity was observed. A random effects model was selected if significant heterogeneity was observed. Possible sources of heterogeneity were explored through a meta-regression analysis with covariates as follows: (1) country (China vs. countries other than China), (2) number of patients (˃ 45 vs. < 45), (3) location (ICU vs. OR), and (4) reference standard device(PiCCO vs. no-PiCCO).

## Results

### Characteristics of the studies

**Fig. 1:**
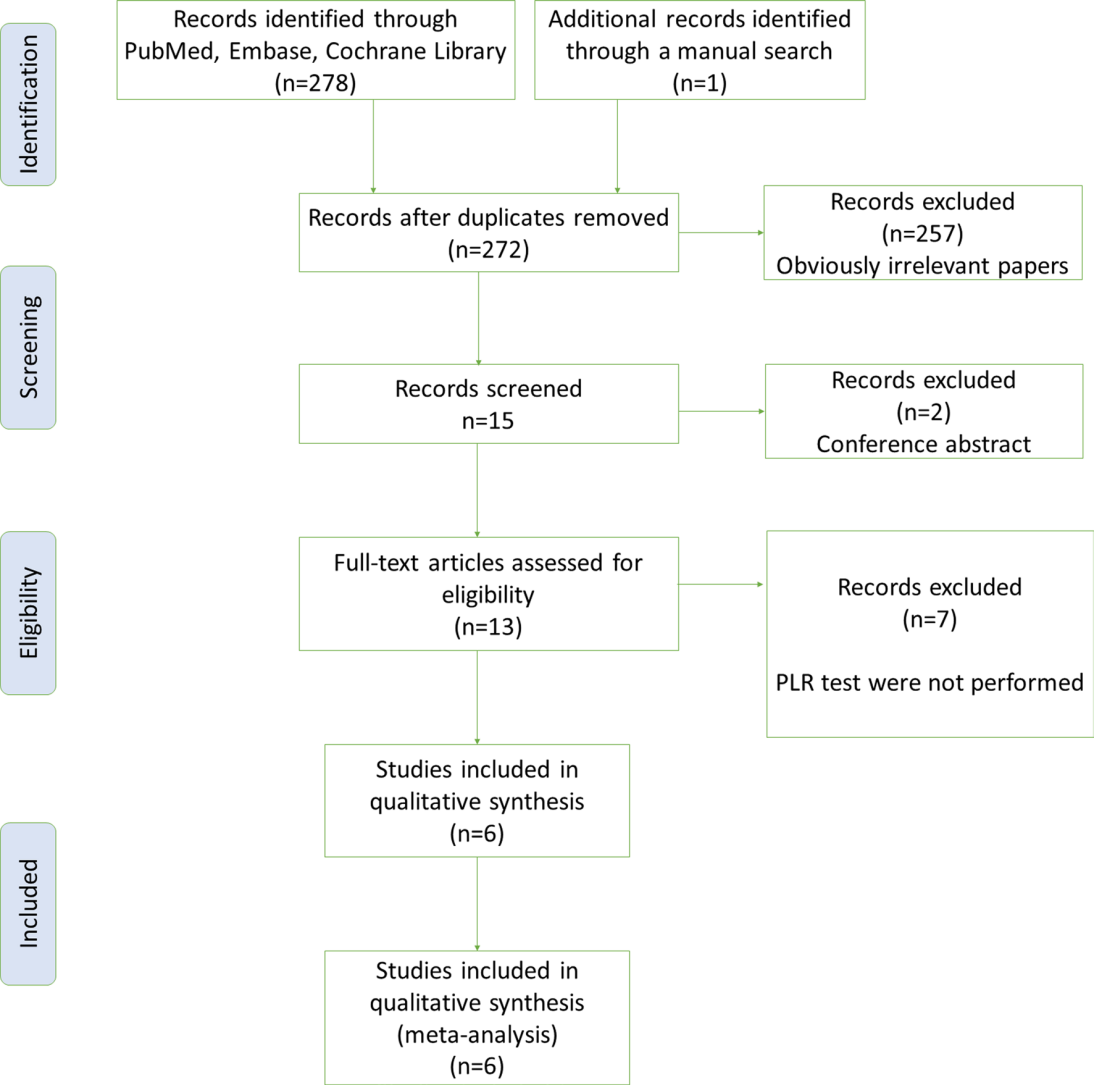
Flow diagram of identification of studies

**Fig. 2:**
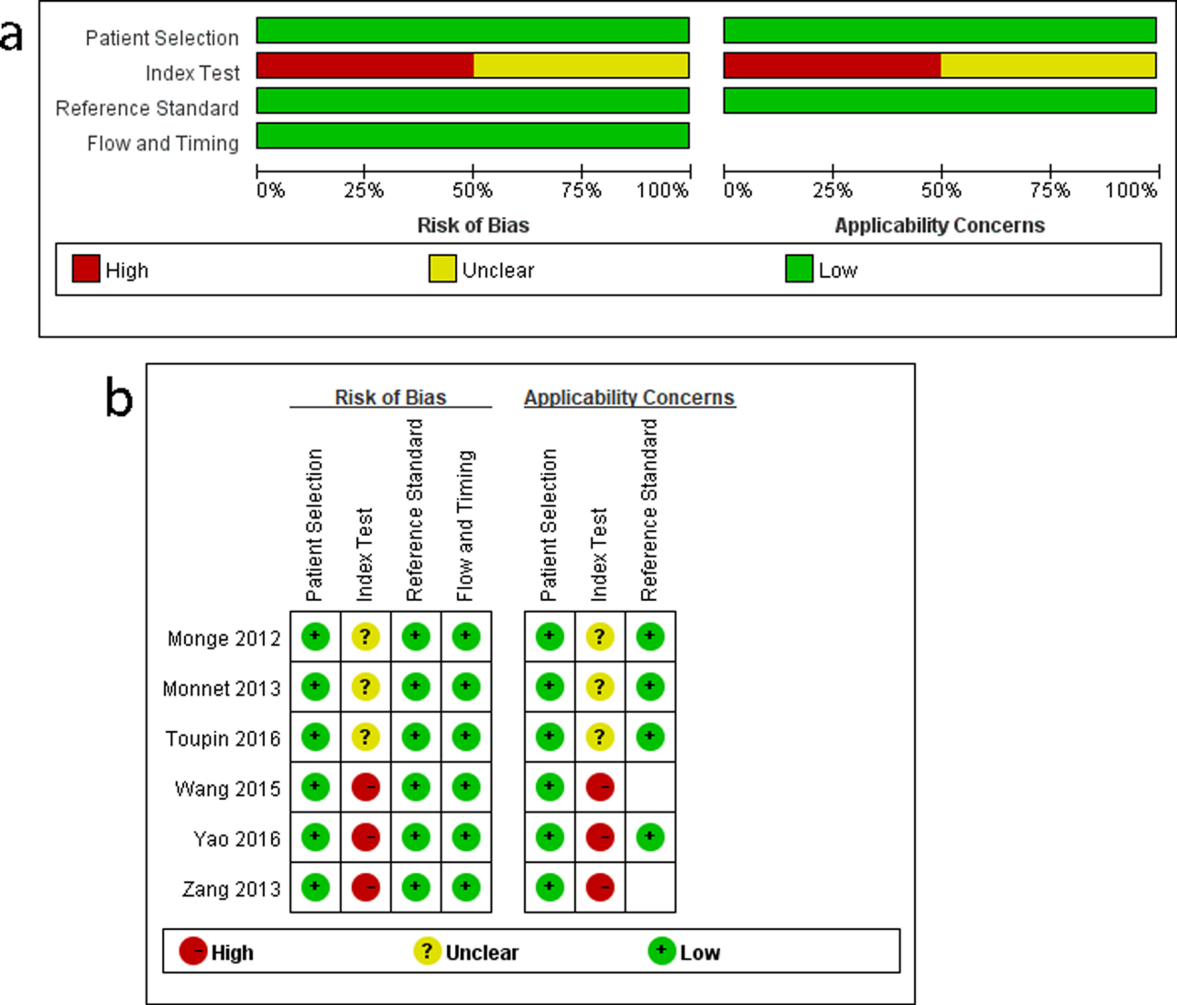
Risk of bias and applicability concerns for the studies included in the meta-analysis. **a** Risk-of-bias graph and **b** risk-of-bias summary

This meta-analysis yielded 279 primary studies after the initial independent review, comprising 278 published studies identified through electronic database searches and one published study identified through a manual search. Figure 1 shows the study selection process. A total of seven records were initially excluded due to duplicate records; 259 records were excluded due to the source not related to the research topic or being conference abstract; and seven records were excluded because PLR test was not performed. Finally, six studies[17, 18, 30–33] fulfilled all the inclusion criteria and were considered for analysis. They are all prospective single-center studies, the main characteristics of the eligible studies are shown in Table 1. The quality of the included studies was assessed using QUADAS-2 available in Fig. 2.

**Table 1:** Characteristics of the studies included in this meta-analysis

First author/year of publication	Country	Location	Patients	Gender (M/F)	Age (year) Mean ± SD	Cases	Tidal volume (mL/kg)	Index test device	Reference standard measurement	Reference standard threshold (%)	Reference standard device
Monge/2012 [18]	Spanish	ICU	Patients with controlled mechanical ventilation and acute circulatory failure	16/21	64 ± 13	37	8.1 ± 1.2	Side stream infrared gas analyzer	CO	≥ 15	Cardio Q
Monnet/2013 [17]	France	ICU	Patients ventilated in the control assisted mode with no inspiratory effort and hemodynamic instability	NA	60 ± 14	40	6.4 ± 0.8	Side stream infrared gas analyzer	CI	15	PiCCO
Zang/2013 [30]	China	ICU	Patients with controlled mechanical ventilation and sepsis shock	22/20	Responder: 56.9 ± 16.6 Nonresponder: 57.7 ± 12.3	42	Responder: 7.5 ± 3.6 Nonresponder: 7.7 ± 2.3	Side stream infrared gas analyzer	CI	≥ 15	PiCCO
Wang/2015 [31]	China	ICU	Patients with controlled mechanical ventilation and sepsis shock	24/24	Responder: 52.7 ± 29.4 Nonresponder: 56.7 ± 26.2	48	NA	Side stream infrared gas analyzer	CI	> 10	PiCCO
Toupin/2016 [32]	Canada	OR	Patients receiving mechanical ventilation and undergoing cardiac or ascending aortic surgery	62/28	Responder: 68 ± 10 Nonresponder: 65 ± 10	90	6–8	Side stream infrared gas analyzer	CI	≥ 15	PAC
Yao/2016 [33]	China	ICU	Patients with controlled mechanical ventilation and shock post-cardiac surgery	27/14	Responder: 55.4 ± 9.9 Nonresponder: 58.5 ± 9.3	41	8–10	NA	CI	≥ 15	PiCCO

CI, cardiac index; CO, cardiac output; SD, standard deviation; ICU, intensive care unit; OR, operation room; Cardio Q, Cardio Q-ODM™ esophageal Doppler monitor. PiCCO, pulse indicator continuous cardiac output; PAC, pulmonary artery catheter; NA, not available

### Quantitative synthesis

**Fig. 3:**
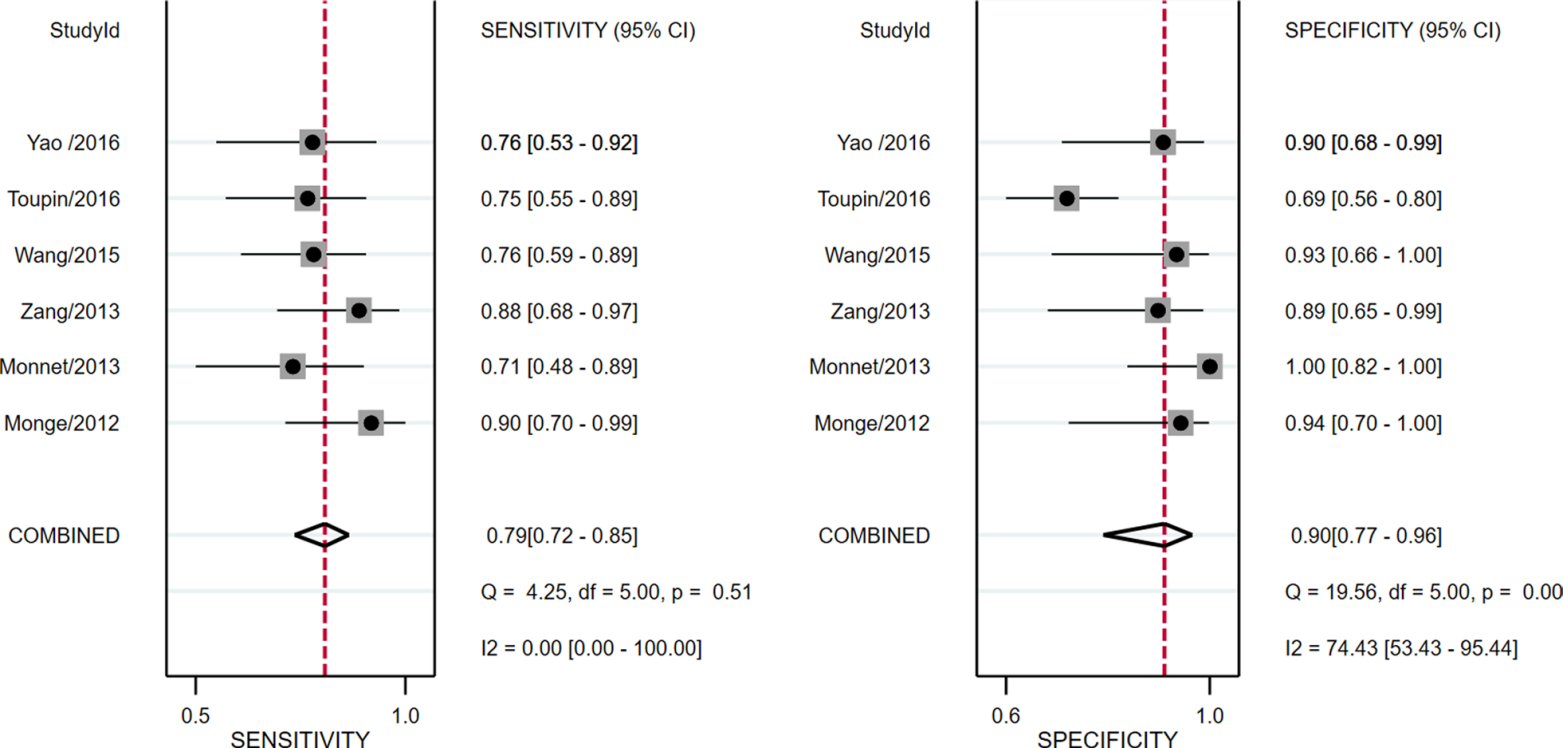
Forest plots of the pooled sensitivity and specificity. Each solid square represents an individual study. Error bars represent 95% CI. Diamond indicates the pooled sensitivity and specificity for all of the studies

**Fig. 4:**
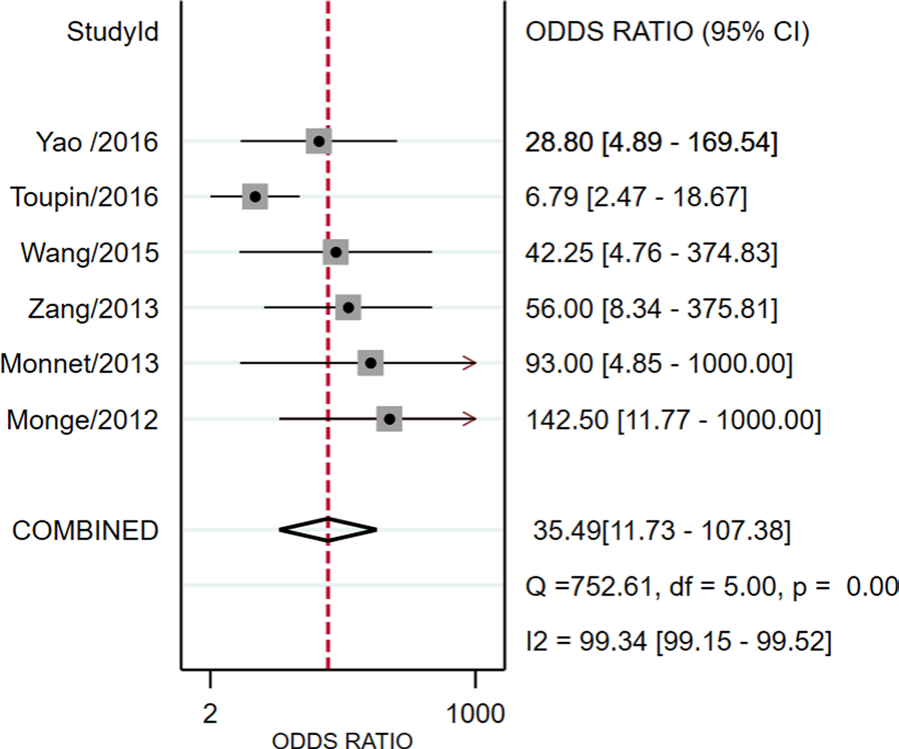
Forest plots of the pooled diagnostic odds ratio. Each solid square represents an individual study. Error bars represent 95% CI. Diamond indicates the pooled diagnostic odds ratio for all of the studies

**Fig. 5:**
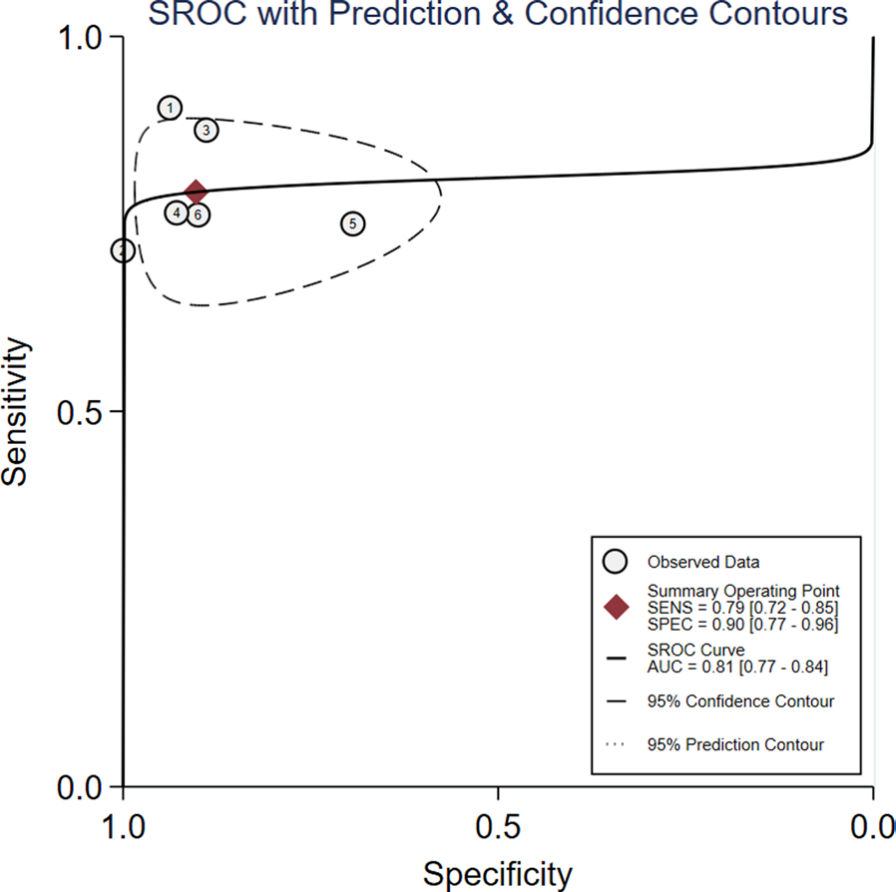
SROC curve of variation of end-tidal carbon dioxide for predicting fluid responsiveness. Each circle represents individual study estimates. The diamond is the summary point representing the average sensitivity and specificity estimates. The ellipses around this summary point are the 95% confidence region (dashed line) and the 95% prediction region (dotted line). The cutoff value of included studies: (1) Monge/2012 [16]: 5%; (2) Monnet/2013 [15]: 5%; (3) Zang/2013 [28]: 5%; (4) Wang/2015 [29]: 5%; (5) Toupin/2016 [30]: 2 mmHg; and (6) Yao/2016 [31]: 5.8%

Study data and individual diagnostic estimates are summarized in Table 2. Overall, 298 patients were included in this review, of whom 149 (50%) were fluid responsive. The cutoff values of ΔEtCO2 in four studies was 5%, one was 5.8% and the other one was an absolute increase 2 mmHg. The AUROC of individual studies ranged from 0.80 to 0.94. Heterogeneity between studies was assessed with an overall *Q* = 4.098, *I*^2^ = 51%, and *P* = 0.064, indicated substantial heterogeneity. Spearman’s correlation coefficient was − 0.6 (*P* = 0.28), indicating no threshold effect. The pooled sensitivity and specificity for the overall population were 0.79 (95% CI 0.72–0.85) and 0.90 (95% CI 0.77–0.96), respectively (Fig. 3). The pooled positive likelihood ratio and negative likelihood ratio were 8.2 (95% CI 3.2–20.5) and 0.23 (95% CI 0.16–0.32), respectively. The DOR was 35 (95% CI 12–107) (Fig. 4). The pooled AUROC was 0.81 (95% CI 0.77–0.84) (Fig. 5).

**Table 2:** Summary of results of the studies included in this meta-analysis

First author/Year of publication	Sample size	Cutoff value (increase in percentage or absolute value)	Subject numbers could be calculated				Sensitivity (%)	Specificity (%)	AUROC (95%CI)
			TP	FP	FN	TN			
Monge/2012 [18]	37	5%	19	1	2	19	90.5	93.7	0.94 (0.82–0.99)
Monnet/2013 [17]	40	5%	15	0	6	15	71	100	0.93 (0.81–0.99)
Zang/2013 [30]	42	5%	21	2	3	21	88	88.2	0.90 (0.775–1.0)
Wang/2015 [31]	48	5%	26	1	8	26	75.8	93.4	0.849 (0.739–0.93)
Toupin/2016 [32]	90	2 mmHg	21	19	7	21	75	70	0.80 (0.70–0.90)
Yao/2016 [33]	41	5.8%	16	2	5	16	76.2	90	0.875 (0.769–0.981)

AUROC, Area under the receiver operator characteristics curve; CI, confidence interval; FN, false negative; FP, false positive; NA, not available; TN, true negative; TP, true positive

### Meta-regression analysis results

**Fig. 6:**
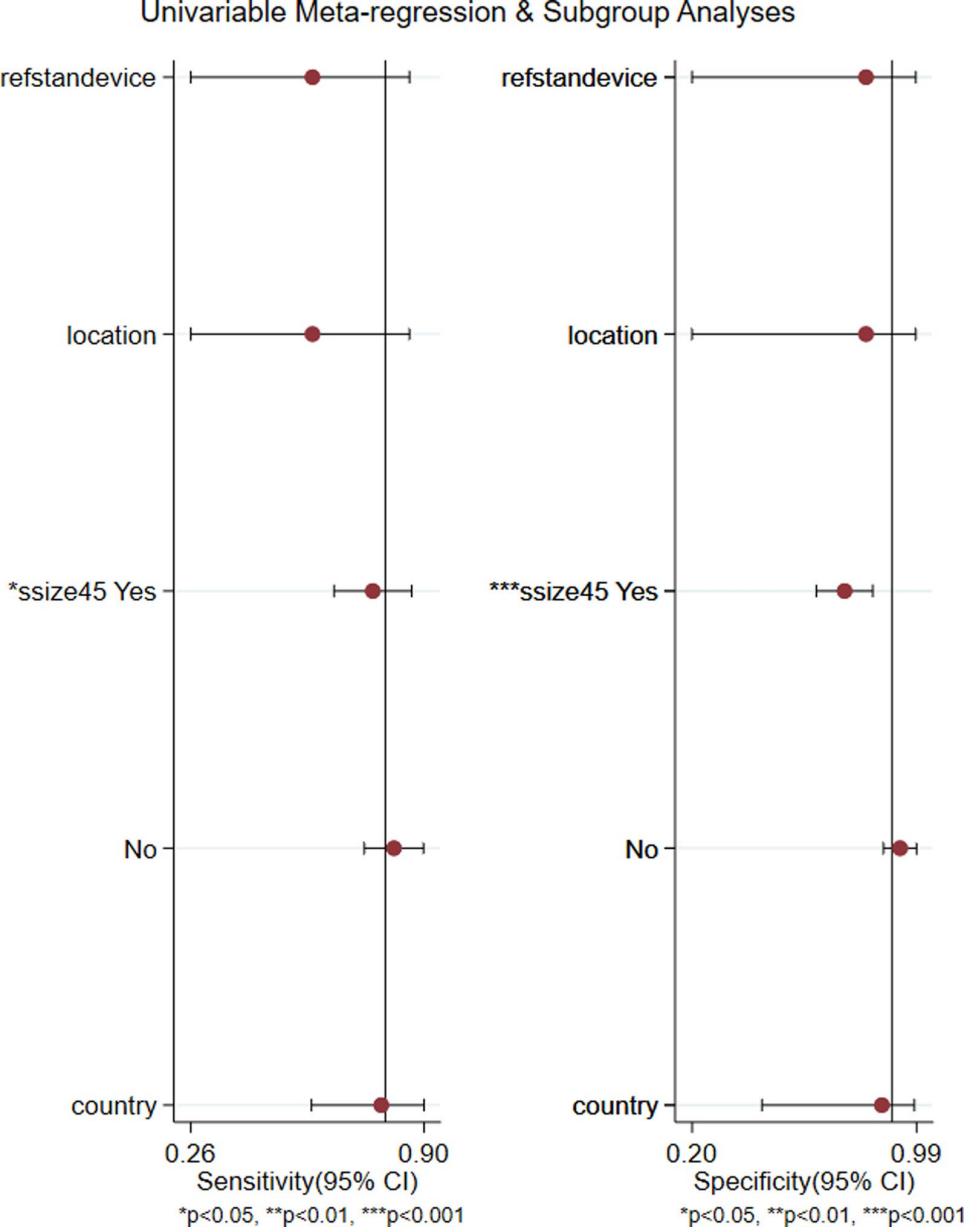
Graphs for meta-regression analysis. CI = confidence interval. Meta-regression was performed by country (China vs. countries other than China), number of patients (˃ 45 vs. < 45), location (ICU vs. OR), and reference standard device(PiCCO vs. no-PiCCO)

The results of the meta-regression analyses (Fig. 6) showed that the significant sources of heterogeneity in sensitivity and specificity were the number of patients larger than 45. Other factors, including country, location, and reference standard device, were not significantly different.

### Publication bias and sensitivity analysis

**Fig. 7:**
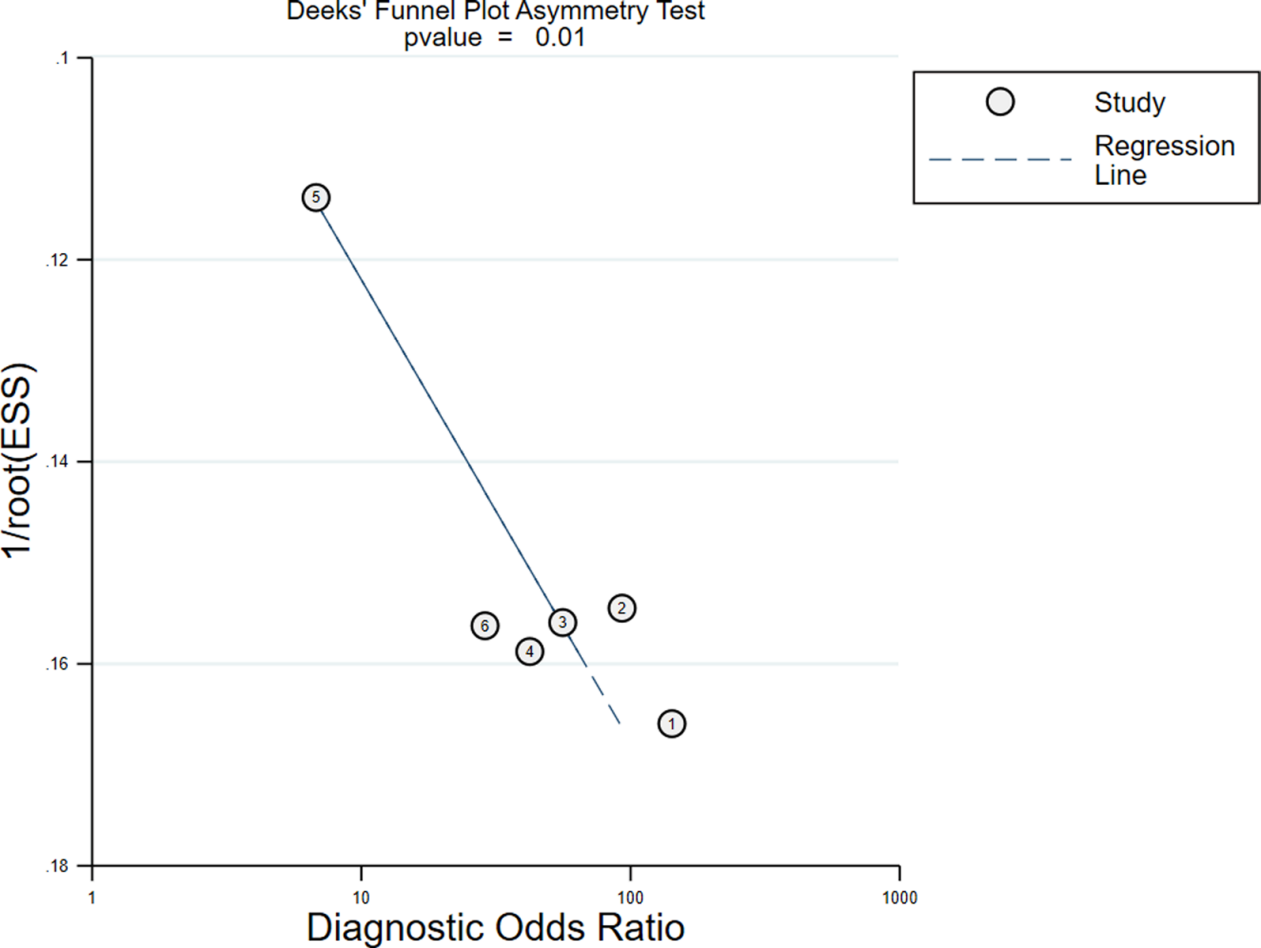
Deeks’ funnel plot of publication bias among studies. ESS = effective sample size. Numbers 1 to 6 represent the study arms (Monge/2012, Monnet/2013, Zang/2013, Wang/2015, Toupin/2016, Yao/2016)

**Fig. 8:**
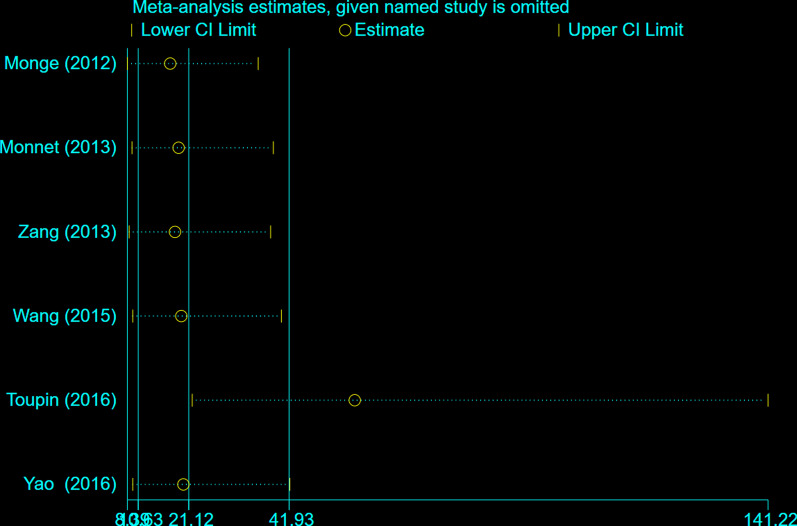
Graphs for sensitivity analysis

The publication bias of the studies was assessed using the Deeks’ funnel plot asymmetry test. The slope coefficient of the six studies was associated with a *P* value of 0.01 (Fig. 7). The aforementioned results indicated significant publication bias. The sensitivity analysis (Fig. 8) showed that excluding Toupin’s study [32] negated the publication bias (*P* = 0.36). The sensitivity analysis also showed that the pooled DOR ranged from 21 to 140 and the pooled AUC ranged from 0.92 to 0.96 when one study was omitted.

## Discussion

The correct evaluation of intravascular volume and proper maintenance of cardiac preload can improve the prognosis of critically ill patients. Static variables could poorly predict fluid responsiveness [34–37]. However, dynamic indicators of fluid responsiveness, which are based on cardiopulmonary interactions in patients receiving mechanical ventilation, have been shown to be predictive [38–42]. The ΔEtCO_2_ has been extensively studied with respect to its value in predicting fluid responsiveness, but the results are conflicting [13–18, 30–33, 43–45]. To the best of our knowledge, this is the first systematic review and meta-analysis to explore the diagnostic accuracy of ΔEtCO_2_ in predicting fluid responsiveness during PLR test in patients with mechanical ventilation. The results confirmed that, overall, the ΔEtCO_2_ performed moderately in predicting fluid responsiveness in patients with mechanical ventilation during PLR test, with a pooled AUROC of 0.81 (95% CI 0.77–0.84), a pooled specificity and sensitivity of 0.90 (95% CI 0.77–0.96) and 0.79 (95% CI 0.72–0.85). These findings are clinically relevant because capnography was widely available for critically ill and surgical patients, and ΔEtCO_2_ values can be obtained immediately in the emergency or critical care setting and operation room.

The PLR test provides a dynamic assessment of preload dependence inducing a transient and reversible increase in cardiac preload. This test has been demonstrated to predict fluid responsiveness in many studies over a wide population, including clinical situations in which other parameters of fluid responsiveness have failed, such as patients with cardiac arrhythmias or with spontaneous breathing [8, 9]. However, the PLR must be interpreted in conjunction with changes in CO, velocity time integral, aortic blood flow velocity, carotid artery flow time [46], the measurement of such index often requiring expensive or invasive hemodynamic monitoring systems, resulting in its wide application is limited.EtCO2 can be easily determined with continuous waveform-capnography devices that are generally available in the clinical. In the present study, we showed that fluid responsiveness can be assessed using EtCO2 monitoring and PLR test. This provides a clinically useful way to predict fluid responsiveness using readily available diagnostic tools such as a PLR maneuver and EtCO2 measurement by capnography.

The present systematic review and meta-analysis had some limitations. First, this analysis included only six studies with a relatively small sample size even pooled analysis there are only 298 patients were included in this review; The 95% CI AUROC is between 0.77 and 0.84 and the studies heterogeneity is great. Therefore, the power and precision of the results were limited. Second, standard PLR maneuver was performed except for Toupin’s study [32], the time for assessing the change in CO and EtCO2 in Zang’s [30] studies was when PLR had induced its maximal effect on CI, other studies were recorded one minute after PLR, this could significantly impact the results. Third, the quality assessment showed a high risk of bias in the index test. This bias might have restricted the interpretation of the true diagnostic efficacy of ΔEtCO2 value in predicting fluid responsiveness. Forth, publication bias was observed among studies. We performed a sensitivity analysis and found that the pooled DOR ranged from 21 to 140 and the pooled AUC ranged from 0.92 to 0.96. This indicates that the results were stable despite the presence of publication bias. Finally, since more detailed individual patient data were not available, a more comprehensive analysis of diagnostic effect could not be conducted.

## Conclusions

The study confirmed that the ΔEtCO_2_ performed moderately in predicting fluid responsiveness in patients with mechanical ventilation during PLR test. As the limited number of studies included and study heterogeneity, further studies with a larger dataset and well-designed models are required to confirm the diagnostic accuracy and utility of EtCO2 in predicting fluid responsiveness in patients with mechanical ventilation during PLR test.

## Data Availability

The datasets used and/or analyzed during the current study are available from the corresponding author on reasonable request.

## Abbreviations

ΔEtCO2: Variation of end-tidal carbon dioxide
PLR: Passive leg raising
EtCO2: End-tidal carbon dioxide
DOR: Diagnostic odds ratio
CI: Confidence interval
TP: True positive
FP: False positive
TN: True negative
FN: False negative
AUROC: Area under the receiver operator characteristic curve
QUADAS-2: Quality Assessment of Diagnostic Accuracy Studies
SROC: Summary receiver operator characteristic
AUC: Area under the SROC curve
